# The Impact of Activity-Based Mobility Pattern on Assessing Fine-Grained Traffic-Induced Air Pollution Exposure

**DOI:** 10.3390/ijerph16183291

**Published:** 2019-09-07

**Authors:** Yizheng Wu, Guohua Song

**Affiliations:** Key Laboratory of Transport Industry of Big Data Application Technologies for Comprehensive Transport, Beijing Jiaotong University, Beijing 100044, China

**Keywords:** air quality assessment, activity-based mobility, dynamic human exposure assessment, population-weighted concentration

## Abstract

Quantifying the air pollution and health impacts of transportation plans provides decision makers with valuable information that can help to target interventions. However, a large number of environmental epidemiological research assumes exposures of static populations at residential locations and does not consider the human activity patterns, which may lead to significant estimation errors. This study uses an integrated modeling framework to predict fine-grained air pollution exposures occurring throughout residents’ activity spaces. We evaluate concentrations of fine particulate matter (PM_2.5_) under a regional transportation plan for Sacramento, California, using activity-based travel demand model outputs, vehicle emission, and air dispersion models. We use predicted air pollution exposures at the traffic analysis zone (TAZ) level to estimate residents’ exposure accounting for their movements throughout the day to assess the impact of activity-based mobility pattern on air pollution exposure. Results of PM_2.5_ exposures estimated statically (at residential locations) versus dynamically (over residents’ activity-based mobility) demonstrates that the two methods yield statistically significant different results (*p* < 0.05). In addition, the comparison conducted in different age groups shows that the difference between these two approaches is greater among youth and working age residents, whereas seniors show a similar pattern using both approaches due to their lower rates of travel activity.

## 1. Introduction

It is of particular importance to assess the air pollution exposure in California. The Assembly Bill (AB) 617, signed into law in July 2017, continues California’s environmental leadership in establishing innovative new policies to improve air quality [1]. The bill requires new community-focused and community-driven action to reduce air pollution and improve public health in communities that experience disproportionate burdens from air pollution. In November 2015, The US Environmental Protection Agency (US EPA) released and updated the final guidance for modeling the local air quality impacts of certain transportation projects on the fine particulate matter (PM_2.5_) national ambient air quality standards (NAAQS) [2]. The proposed modeling chain includes an air dispersion modeling to obtain ambient pollution concentration levels but does not include an evaluation of people’s exposure to the pollutants. In light of the considerable epidemiological evidence to suggest a relationship between exposure to PM_2.5_ and negative health effect [3,4,5], it is necessary to incorporate the human exposure assessment into air quality evaluation.

Integrated models of travel demand, emissions, and air dispersion are a promising direction for allowing planners to evaluate the air quality and human exposure for different groups and in different regions. They overcome the limitation of air pollution data sourced from a small number of fixed-site monitors. The modeling framework has been used to estimate existing transportation pollution concentrations at fine spatial scales [6,7], as well as to forecast the air quality effects of transportation plans and policies at fine scales [8]. Some existing fine-grained integrated modeling studies go further by forecasting the health effects of transportation policies [9] and plans [10]. Rowangould et al. [9] quantified the health effects of PM_2.5_ due to the revision of a clean truck program at the Port of New York and New Jersey by integrating vehicle activity with the emissions, dispersion, and health models. Poorfakhraei et al. [10] applied an integrated modeling framework to evaluate the health impacts due to the implementation of a long-range transportation plan in Atlanta. However, both studies overlay US census block-level population estimates (which reflect static residential locations) with corresponding air pollution concentrations, essentially assuming a static population distribution in the exposure assessment. Assuming a static population is also common in epidemiological studies (e.g., [3,11,12]). This type of approximation may be less problematic when comparing exposures in different regions (where a region’s entire population is assumed to be exposed to an average regional concentration). However, for finer scale analyses that compare population or community exposures within a region, using residents’ home locations to approximate their activity space fails to account for their exposure as they travel to work, school, etc., likely over- or under-estimating their risks when these destinations coincide with lower or greater concentrations than residents’ homes, respectively. A handful of public health studies do account for residents’ movements throughout the day [13,14] but the spatial scale in these studies may still be too coarse to capture localized variations in pollution in urban areas with a complex exposure profile. Some researchers apply the new technology (mobile devices) to track the residents’ location (e.g., [15]), however, this data source is not easy to obtain and also has privacy concerns. For transportation agencies, quantification of traffic-related air pollution for transportation plans and projects is essential, and many typically use the activity-based models (ABM) which represent peoples’ activity pattern. Hence, the air quality assessment accounting for human mobility is feasible during the evaluation process for many transportation agencies.

The aim of this study is to assess the population-weighted concentration of traffic-induced PM_2.5_ in a US city at a fine-grained spatial scale (traffic analysis zone, TAZ level) while accounting for residents’ mobility pattern to demonstrate its impact on zonal air quality evaluation. The population-weighted concentrations are estimated by incorporating people’s activity (extracted from an ABM) and PM_2.5_ concentrations (estimated using an integrated modeling framework that includes an activity-based travel demand model and vehicle emission and air dispersion models). These estimates can identify specific locations where elevated pollution concentrations and high numbers of people overlap, which can be used to identify particular locations that may be targeted for emissions reductions. We also compare the estimated air pollution level derived from static (assuming people are always located at their home location) and dynamic (considering people’s activity) population exposures for different age groups with various activity patterns. It is intended to introduce an air quality assessment index (population-weighted concentration) accounting for human activity patterns for potential application in future environmental impact assessment of transportation plans.

## 2. Methods and Data

This study assesses the air quality impacts of the Sacramento Area Council of Governments’ (SACOG’s) 2016 Metropolitan Transportation Plan/Sustainable Communities Strategy (MTP/SCS) for the City of Sacramento, California. It is tested using simulated travel behavior data estimated by SACOG’s ABM (Sacramento, CA, U.S.), SACSIM15, which underlies their 2016 MTP/SCS [16].

Pollution concentration levels for the baseline and scenario were quantified at a fine spatial scale by well-calibrated modeling tools and expressed as population-weighted concentrations to represent the effects on a dynamic population distribution. The detailed framework of air quality assessment is presented in next section.

### 2.1. Air Quality Modeling Framework

This study uses a chain of modeling components (from traffic demand to vehicle emissions to air dispersion) to derive a spatially disaggregated estimate of pollution concentrations. Further, it is essential to evaluate the ultimate effects of air pollution on human health by accounting for people’s activity pattern. The model framework applies the human mobility information from the local ABM (SACSIM15) to overcome the limitations of using static residential census data by providing dynamic information on the time activity pattern and location of individuals. Accounting for the physical location of the population is critical when evaluating the effects of vehicle emissions because vehicular pollution concentrations can vary widely across a region.

**Figure 1:**
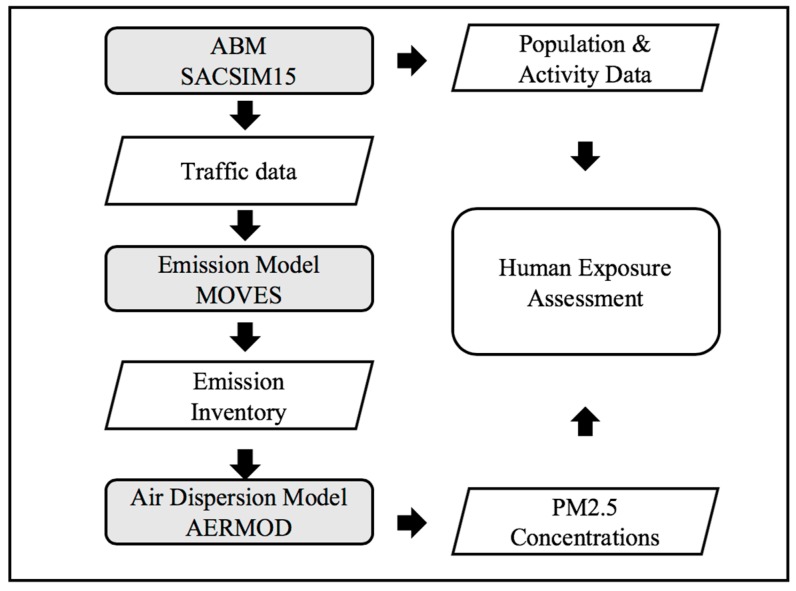
Overall dataflow of air quality model framework (grey rectangles represent models and white parallelograms represent data sets).

The air quality modeling framework includes the local ABM, emission model, and air dispersion model (Figure 1). In this research, SACSIM15 is used as the local ABM, MOVES (version 2014a) is applied as the emission model, and AERMOD (version 16216r) is applied as the air dispersion model. The latter two are regulatory models that reflect the state of art and practice. Although in California the regulatory emission model is EMFAC, since MOVES is used more widely across the nation, this study applies MOVES as the emission model to demonstrate the general application of the modeling framework.

SACSIM15 outputs the trip information for each individual in its synthetic population, which is used to identify the individual location and activity pattern in the exposure assessment. In addition, the model also provides a loaded traffic network for the SACOG region, including the traffic volume and average speed for each roadway link. By incorporating the traffic information from SACSIM15, MOVES is run in the “Emission Inventory” scale to produce composite emission rates in terms of grams/hour (g/h). After running the post-processing script for PM_2.5_ in terms of g/h, the emission rates for each link are extracted from the MySQL database from MOVES and passed on to the air dispersion model to estimate concentrations at desired locations. AERMOD is used to estimate the concentration of PM_2.5_ based on meteorological conditions, roadway locations, and emissions rates.

### 2.2. Human Exposure Assessment

The population-weighted air pollution concentration is estimated by combining population activity distribution and duration information from SACSIM15, and the TAZ-level concentration is estimated using AERMOD.

The human exposure assessment is evaluated at the TAZ level, which is an appropriate scale for tracking the dynamic population distribution. For each spatial unit (i.e., TAZ), the hourly population data and concentration data are combined for each hour of the day to quantify the population-weighted average concentration, as shown in the following equation:

(1) $${\bar{C}}_{i}=\frac{\sum_{h=1}^{24}P_{i}\left(h\right)C_{i}\left(h\right)}{\sum_{h=1}^{24}P_{i}\left(h\right)}$$

where, ${\bar{C}}_{i}$ is the population-weighted average concentration level (in the unit of µg/m^3^) for location *i*, $P_{i}\left(h\right)$ is the population counts for hour, *h,* at location *i*, and $C_{i}\left(h\right)$ is the PM_2.5_ concentration level (in the unit of µg/m^3^) for hour, *h,* at location *i*. This index considers the individual activity and spatial-temporal concentration variation of each location.

### 2.3. Case Study Setup

**Figure 2:**
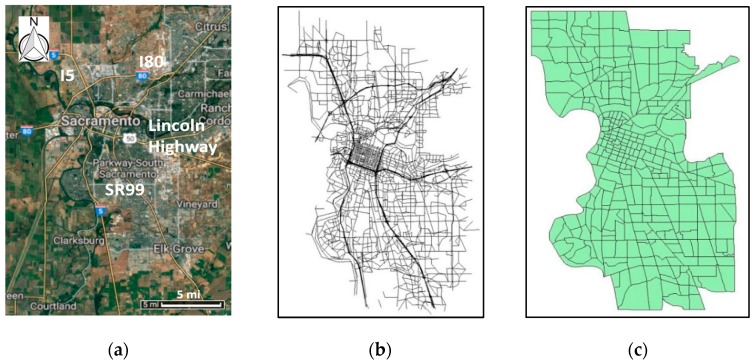
Sacramento study area. (**a**) Case study domain (source: Google Map), (**b**) Roadway network in Baseline 2012, (**c**) traffic analysis zones (TAZs).

The modeling domain is the City of Sacramento, the capital of California, with a 2016 population of 495,234 according to the US Census Bureau. It has a total area of 100.1 square miles, which are modeled as 334 TAZs (averaging about 0.3 square miles) in SACSIM15 (Figure 2). Several major traffic corridors pass through Sacramento, including Interstate Highway 5 (I5), Interstate Highway 80 (I80), State Route 99 (SR99), and the Lincoln Highway.

This study uses simulated travel behavior data estimated by SACSIM15 under the 2016 MTP/SCS. The plan must meet the requirement of Sustainable Communities and Climate Protection Act of 2008 (commonly known as SB 375) requiring per-capita reductions in future greenhouse gas emissions resulting from changes in travel behavior [17]. SACOG has used SACSIM15 to estimate changes in travel behavior for the present and three alternative scenarios under the 2016 MTP/SCS. The plan consists of a baseline in 2012 and three alternatives in the future year 2036. In this study, we select baseline 2012 and one alternative scenario 2036 (S2) to analyze the air pollution effects of the implementation of transportation plan. The main characteristics of the scenario are listed in Table 1.

In the 2012 baseline year, there are over 1600 miles of major roadways represented by 6588 links in SACSIM15. The future scenario uses the network with over 1800 miles represented by 7354 links. The travel demand model estimates the traffic counts for four time periods, morning peak (AM, 6 a.m. to 9 a.m.), mid-day (MD, 9 a.m. to 4 p.m.), evening peak (PM, 4 p.m. to 7 p.m.), and overnight (ON, 7 p.m. to 6 a.m.). In addition, the average vehicle speed, lane numbers, and link length are also provided by the model. Then the traffic data pass to MOVES to estimate the PM_2.5_ emission rates for each link in each time period.

#### 2.3.1. Estimates of Traffic-Induced Emissions

MOVES is used to estimate the emission rates for each link and time period. Sixteen model runs (four seasons and four time periods) are used to estimate the annual average emission rates in each of the two scenarios (2012 baseline and 2036 scenario), resulting in a total of 32 model runs. January, April, July, and October were chosen as the representative months for each season. Meteorological files from the surface station at Sacramento International Airport, which is the closest station to the modeling domain, were obtained from the California Air Resources Board (CARB). This meteorological data is used to determine the temperature and humidity required to calculate emissions. Meteorological conditions are assumed to remain the same for the 2012 and the 2036 scenario.

In MOVES, PM_2.5_ emissions are estimated for vehicle exhaust, crankcase running exhaust, brake wear, and tire wear. The result is the composite emission rates (g/h) for each roadway link. Those links are then used as emission sources in the AERMOD model.

#### 2.3.2. Estimates of Air Pollution Concentrations

The Sacramento International Airport meteorological data obtained from CARB are also used to estimate pollution dispersion. The processed hourly meteorological data (including temperature, humidity, wind, etc.) and roadway information for each link (including emission rates, longitude/latitude, angle, width, and length) are incorporated into AERMOD in the form of external files.

Emissions from each link are traced to user-specific point virtual receptor locations in AERMOD. The dispersion of emissions from each link to each receptor is modeled independently by AERMOD. Receptors are placed at an average human inhalation height of 1.5 m. Since the pollutant concentration varies along roadways [7,18], to capture this heterogeneity, receptors were spaced at distances of 100 m within 1000 m of the major corridors and downtown area, with 200 m spacing for the rest of the case study domain (resulting in a total of 18,175 modeled receptors). Because seasonal variation can be processed by using a seasonal factor in the AERMOD setup, there are at total of 8 model runs for the two scenarios and four time periods. The results are the annual average PM_2.5_ concentrations (µg/m^3^) for each receptor. Because the spacing between receptors varies in some TAZs, spacing-weighted average concentration levels are estimated for each TAZ after geocoding receptors into TAZs.

#### 2.3.3. Extraction of Activity Pattern

Individual trip information is obtained from SACSIM15, including the origin TAZ and destination TAZ for each trip, departure time, and arrival time. Sample data are shown in Table 2, which represents an individual’s entire daily trip pattern.

Trips for the residents of Sacramento are identified and divided into age-gender categories. The population in each TAZ is estimated for each hour and each age-gender category. Every person’s location is identified as the TAZ with the highest fraction of time spent in a given hour. Records with abnormal time information are excluded, i.e., departure/arrival time is negative or greater than 24:00 (about 0.5% of the total records). Total trip numbers are 2.01 and 2.67 million for the baseline (2012) and scenario (2036), respectively. The calculation is performed using the R statistical computing language [19]. The five main steps are illustrated below using the example data in Table 2:

1. Extract the trip chain for each individual. For example, Table 2 is a complete trip chain for a person. The trip chain is allocated into an age-gender category based on the traveler’s demographics.

2. For the first trip, from 00:00 to the departure time, the person’s location is recorded as the origin TAZ. In Table 2, from midnight to 4 p.m., the person’s location is TAZ 1240.

3. For the last trip, from the arrival time to 24:00, people’s location is recorded as the destination TAZ. In Table 2, from 7 p.m. to midnight, the person’s location is TAZ 1240.

4. For other trips, the duration in each TAZ is obtained by subtracting the arrival time of the last trip and the departure time of current trip, and the location is recorded as the origin TAZ of the current trip. In Table 2, from 4 p.m. to 7 p.m., the people’s location is TAZ 1347. Since the duration time in TAZ 1246 is only 7 min, it is not recorded.

5. Once steps 1 to 4 have been completed for all individuals, the final step is to count the population in each TAZ by age-gender category for each hour. The result is output as a matrix, as shown in Table 3.

One limitation of this approach is the missing travel route information at the start and end of each trip. In other words, individuals’ locations are not included when they are traveling between TAZs. The average travel time for each one-way trip is about 20 min for all residents in the City of Sacramento, with residents taking an average of 3.8 trips per day. Given that on-road concentrations are likely to be higher on busy roads, exposure along the travel route may affect estimated health impacts in a dynamic exposure assessment. Further research is required to quantify these potential impacts.

After obtaining the population distribution and the pollution concentration, Equation (1) is applied to compute the population-weighted pollution exposures for both the baseline and scenario. Note that the resolution of traffic volume is aggregated to four time periods, so this analysis assumes that the average hourly concentration in each time period (obtained from AERMOD) applies to all hours in that period.

## 3. Results and Discussion

### 3.1. Vehicle Emission Rates

**Figure 3:**
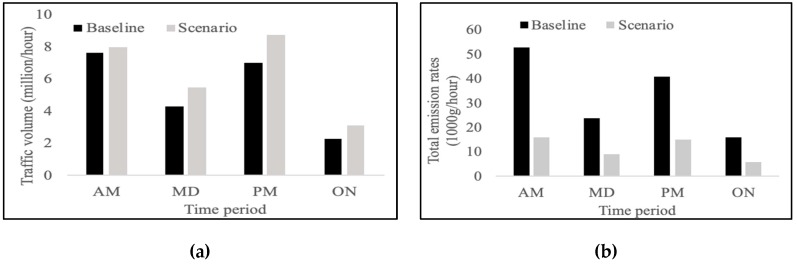
Results of vehicle emissions. (**a**) Total hourly traffic volume, (**b**) Total emission rates.

Figure 3a and b show the total hourly traffic volume and estimated total emission rates (average value across four seasons) respectively, in the roadway network. The 2012 baseline has lower traffic volumes (average 5.30 million/h) and the greater emission rates (average 33.45 kg/h) compared with the 2036 scenario (average 6.32 million/h, 11.42 kg/h), which is likely caused by the increasing stringency of vehicle emission standards and the different structure of the vehicle fleet. As expected, the morning and evening peaks have greater traffic volumes and emission rates than the mid-day and overnight periods. The calculation of emission rates is applied as the emission source in the air dispersion modeling process which demonstrates that the emission estimation is the start of the modeling chain. Hence, a careful choice of modeling tool and modeling process is essential for an accurate estimation result.

### 3.2. Air Pollution Concentrations

**Figure 4:**
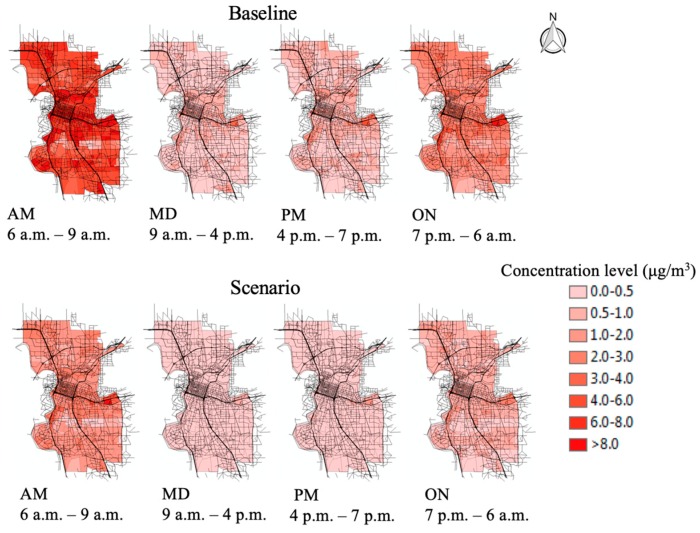
TAZ-level particulate matter (PM_2.5_) concentrations.

Air pollution dispersion modeling results (by time of day and scenario) are shown in Figure 4. Seasonal variations are coded in the model using a seasonal factor. Therefore, the results are annual average concentrations for each TAZ. Concentrations are higher near major corridors like Lincoln Highway and I5. In addition, the maps show significant temporal variation due to the combination of vehicle emission rates and meteorological conditions. Morning peak and overnight have higher pollution concentrations than other periods. Although the evening peak has greater emission rates, the concentrations are generally lower than in the overnight period due to reduced atmospheric mixing overnight and during the morning peak, which leads to higher pollution concentrations. It appears that meteorological conditions play a greater role than emission rates in pollution differences between time periods when looking at the baseline and scenario. Such temporal variation is also observed in other studies [7,20]. The 2012 baseline has the higher concentrations, ranging from 0.003 to 16.55 µg/m^3^ and the averages in each period are 6.41, 0.48, 0.64, and 2.16 µg/m^3^, respectively. Scenario 2036 has a lower upper bound from 0.003 to 8.18 µg/m^3^ and the averages in each period are 1.80, 0.17, 0.26, and 0.72 µg/m^3^, respectively. The difference between baseline and scenario is caused by the traffic-induced emission. The roadway network remains almost the same, but the concentration level shows differences due to the traffic emission rates estimated by the emission modeling process. In Figure 4, we also observed that the high concentration TAZs typically locate close to the major corridors that are considered as the main emission sources in this research.

### 3.3. Population Distribution

**Figure 5:**
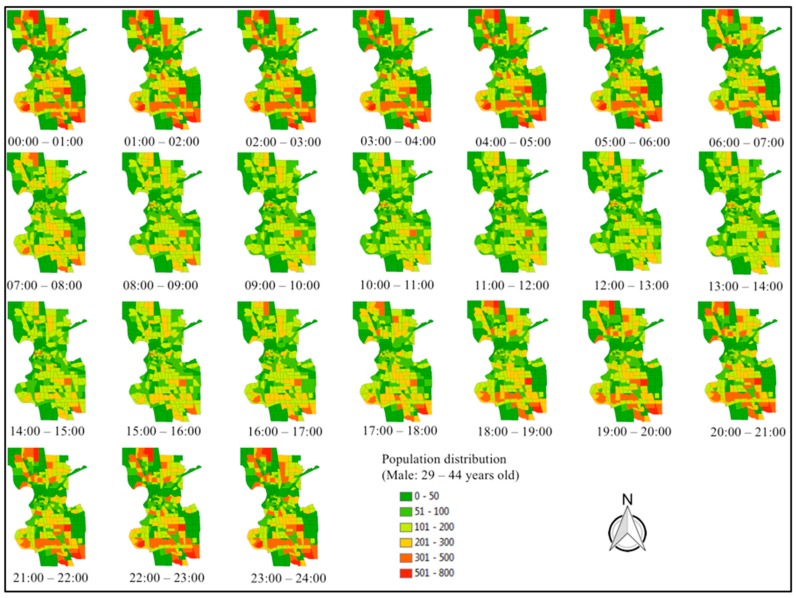
Hourly distribution of population (baseline 2012, male, 29–44 years old).

Using the SACSIM15 trip table, residents’ locations are identified and summarized for all age-gender groups and all scenarios. For example, Figure 5 shows the hourly population distribution in baseline 2012 for males between 29 and 44 years old. Since this group is working age, the locations change considerably during the peak hours and are relatively stable during the night. The population-dense TAZs (in red) during the night are more residential than the rest of the City. The distribution of human activity is also a result from the land use pattern of the city. It should be noted that SACSIM represent all the residents in the SACOG region, therefore, the results in Figure 5 include those people who live outside but stay some time in the City.

### 3.4. Comparison between Dynamic and Static Population Exposure

In the above analysis, the population’s activity space is estimated using the local ABM—SACSIM15. Residents’ locations throughout the day are then combined with PM_2.5_ concentrations to dynamically analyze human exposure to air pollution. Traditionally, approaches to quantifying human exposures (e.g., [7,21]) have relied on static population distributions (i.e., using residential location) by assuming that people stay at their home for the entire time period, which is likely the primary source of the estimation error [22].

In order to better understand the estimation error associated with static population assumptions, we compare the dynamic analysis above to a static analysis (using the same case study, but assuming a static population distribution based on residential location) by using the population-weighted concentration (Equation (1)). In this section, we use the baseline 2012 as an example to illustrate the comparison.

**Figure 6:**
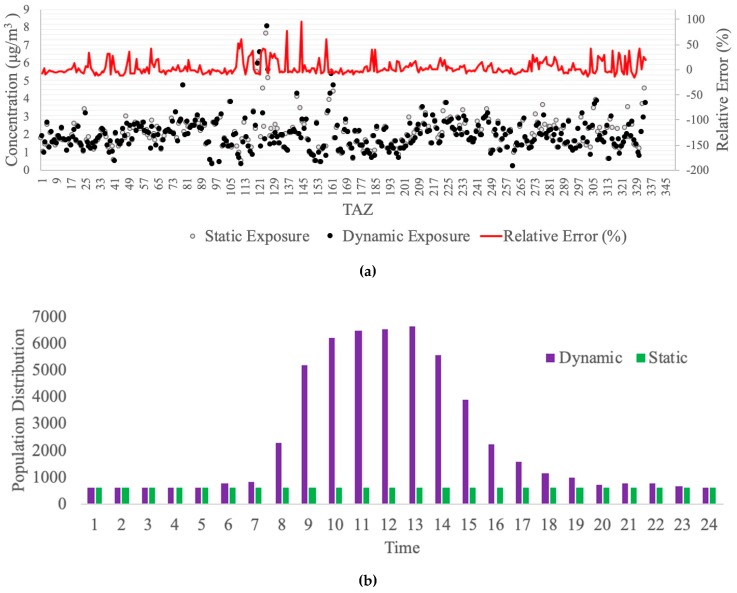
Comparison between dynamic and static estimation. (**a**) Comparison of population-weighted concentration (all TAZs), (**b**) Comparison of population distribution (TAZ 144).

Figure 6a shows the comparison results of these two kinds of concentration levels (dynamic versus static). The relative errors (the ratio of the difference between static result and dynamic result to the dynamic result) range from −15% to 94%. The paired t-test is conducted to check the concentration difference. A criterion of 0.05 for the *p*-value is used, that is, if the *p*-value is smaller than 0.05, then the hypothesis was rejected (i.e., true difference is not equal to 0). The statistic results show the *p*-value equals 2.215e^−4^ (95% confidence interval = (0.024, 0.077)), which means they are significantly different, and the activity-based mobility pattern does make an impact on the air pollution exposure for each TAZ.

We select all TAZs with relative error larger than 50% (TAZ109, TAZ111, TAZ136, TAZ144, TAZ158) and check their land use pattern. All of them consist of trip attractions (workplace, shopping mall, etc.) and few residential areas, resulting that the population distributions show low values in the over-night period and high values in the mid-day period. This real dynamic distribution is obviously different from the simple static assumption, which leads to an estimation error in the evaluation process. We selected TAZ 144 as an example to illustrate the difference of population distribution between two methods (see Figure 6b). TAZ 144 is in the downtown area of Sacramento City and located near several workplaces and schools. There is no residential area in this TAZ. The land use pattern explains the peak form of population distribution extracted from the SACSIM 15. In the mid-day period, it has a large value (more than 5000 people in the TAZ) and has a small value in the over-night period (around 200 people in the TAZ). Obviously, the assumption based on residential location could lead to a significant error in human exposure assessment due to the people’s mobility pattern.

**Figure 7:**
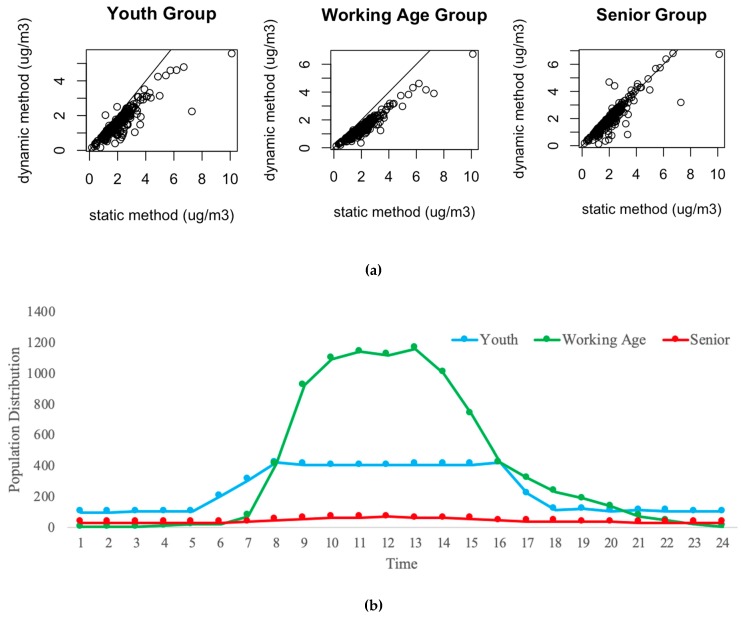
Comparison between dynamic and static method in different age groups. (**a**) Comparison of population-weighted concentrations (all TAZs, the black line is y = x), (**b**) Comparison of population distribution in different age groups (TAZ 144).

Since age is strongly correlated with the health outcomes [23] and different age groups typically represent different activity patterns, we also conduct the comparison for three age categories: youth (4–14 years old), working age (29–44 years old), and seniors (69–79 years old). The resulting population-weighted concentrations are shown in Figure 7a. The results show that for the youth and working age groups, the exposures estimated using the static population distribution are greater than for the dynamic population distribution. The overall differences are 163 and 212 µg/m^3^, respectively. For seniors, the result from the static method is 28.79 µg/m^3^ greater than the one from the dynamic method. A statistical analysis was performed to check the difference in each age group. An ANOVA test and Tukey’s Honestly Significant Difference (HSD) test were applied with a criterion of 0.05 for the *p*-value. The results of the *p*-value are 0.00, 0.00, and 0.58 for youth, working age, and seniors respectively, which demonstrates that the two methods are significantly different in youth and working age groups, but not significantly different in seniors. The relative similarity of the senior values is due to their lower rates of travel activity.

We also select TAZ 144 as an example to show the population distribution in 24 h (see Figure 7b). The senior group shows a relatively stable population number throughout the day. However, the TAZ attracts a large number of working age adults in the day time due to the land use pattern discussed above. There are several schools in TAZ 144 which may cause the number of the youth group to increase during the day. Therefore, for the senior group, the static method may not make a large impact on the results, but for working age and youth groups, it will definitely cause estimation errors due to the active travel pattern.

This comparison section demonstrates that dynamic exposure estimates differ from the more traditional method of assuming a static population. Accounting for the movement of people and their individual activity-space is crucial to accurately quantify human exposure to air pollution.

## 4. Conclusions and Future Directions

In this study, the human exposure to air pollution was evaluated at a fine-grained spatial scale (i.e., TAZ level), accounting for residents’ locations throughout the day to assess the impact of mobility pattern on exposure evaluation. A local transportation plan was evaluated by using the proposed model framework and human exposure assessment in the City of Sacramento, California.

We presented modeling results in each step, including the emission rate estimation, air quality dispersion, and human activity pattern, in order to illustrate the difference between traditional static exposure assessment and dynamic exposure assessment. The final results of the comparison showed that the population-weighted concentration level considering human mobility was significantly different from the traditional static assumption (the *p*-value equals 2.215e^−4^ (95% CI = (0.024, 0.077)). We select one TAZ (TAZ 144) as the example to demonstrate the difference of population distribution between two methods. The dynamic method using the population distribution varies across the day (from 589 to 6616) and the static method only uses 600 to represent the population count for the whole day. Therefore, the dynamic exposure assessment, which considers residents’ movement throughout the day, yields more realistic estimates than static exposure assessments which assume that residents remain at home for the entire day. In addition, the comparison conducted in different age groups show that the difference between these two approaches is greater for youth and working age residents (163 and 212 µg/m^3^, respectively), whereas seniors show a similar pattern (28.79 µg/m^3^) using both approaches due to their lower rates of travel activity. A strict statistical analysis by using ANOVA and Tukey’s HSD was applied to check the differences, and the *p*-values (0.00, 0.00, and 0.58, respectively) also support the conclusion discussed above.

In this study, we account for exposure at residents’ origins and destinations but not along their travel routes, some of which may have elevated pollution levels. Future studies could evaluate the impact of this simplification. We do not have the land use information for the whole region, which may be helpful to analyze the human activity for the whole area. Future study could extract the land use pattern of the case study and conduct a more detailed analysis for the spatial and temporal distribution for both air pollution and human mobility. Additionally, this study estimated primary PM_2.5_ emissions and dispersion and did not account for secondary PM_2.5_ formation.

## Figures and Tables

**Table 1:** Description of the 2016 Metropolitan Transportation Plan/Sustainable Communities Strategy (MTP/SCS) Scenario from Reference [17].

Transportation Inputs	Scenario
New or expanded roads (lane miles, percent increase from 2008)	31%
Transit service (vehicle service hours, percent increases from 2008)	88%
Funding for maintaining and operating the transit system ($ in billions)	$7.9
Funding for new or expanded bus and light rail lines ($ in billions)	$3.4
Funding for bike and pedestrian routes and trail improvements ($ in billions)	$2.8
Additional miles of bicycle paths, lanes and routes	1100

**Table 2:** Sample trip data from SACSIM15.

Sample Number	Person Number	Trip Number	Origin TAZ	Destination TAZ	Departure Time	Arrival Time
1	1	1	1240	1347	16:05	16:12
1	1	2	1347	1246	18:46	18:50
1	1	3	1246	1240	18:57	19:01

**Table 3:** Sample output of population distribution.

Population Counts (Male; 15–29 Years Old)	Hour 1	Hour 2	Hour 3	…
TAZ 1	509	509	508	…
TAZ 2	687	687	687	…
TAZ 3	982	982	980	…
…	…	…	…	…
